# Mind-body-medicine and comprehensive lifestyle-modification in patients with Crohn's disease—Feasibility of a randomized controlled trial under pandemic circumstances

**DOI:** 10.3389/fnint.2022.960301

**Published:** 2022-08-23

**Authors:** Nina Bauer, Claudia Löffler, Özlem Öznur, Christine Uecker, Thomas Keil, Jost Langhorst

**Affiliations:** ^1^Department of Internal and Integrative Medicine, Sozialstiftung Bamberg, Bamberg, Germany; ^2^Department of Integrative Medicine, Medical Faculty, University of Duisburg-Essen, Bamberg, Germany; ^3^Department of Internal Medicine II, University Hospital of Würzburg, Würzburg, Germany; ^4^Institute of Clinical Epidemiology and Biometry, Julius Maximilians University of Würzburg, Würzburg, Germany; ^5^State Institute of Health, Bavarian Health and Food Safety Authority, Erlangen, Germany; ^6^Institute of Social Medicine, Epidemiology and Health Economics, Charité – Universitätsmedizin Berlin, Berlin, Germany

**Keywords:** Crohn's disease, lifestyle modification, mind-body medicine, pandemic, inflammatory bowel disease, stress management, rural conditions, feasibility

## Abstract

**Introduction:**

Mind-body medicine (MBM) focuses on stress reduction and lifestyle changes. The primary objective of this pilot trial was to test study feasibility of a complex integrative MBM program for patients with Crohn's disease (CD), especially in rural regions, and under pandemic conditions.

**Methods:**

Patients were stratified and randomized to the intervention group (IG) or the control group (CG). The intervention included a weekly 6-h session for 10 weeks. The CG (waiting list) received an initial 90-min workshop and started the intervention 9 months later. The primary outcome for study feasibility was recruitment and retention rates, as well as reasons for drop-out. The trial took place in Bamberg, Germany (September 2020 to December 2021).

**Results:**

Totally 700 members of the German Crohn's and Colitis Organization—DCCV—were contacted. A total of 15% (102/700; 95% CI 12–17%) expressed interest to participate. Following screening, 41% (95% CI 32–50) were randomized to IG (*n* = 22) and CG (*n* = 20). The patients were on average (±standard deviation) 48 ± 13 years old, 67% were female, and have been suffering from CD for 20 ± 12 years. Patients traveled 71.5 ± 48.7 km (range: 9–227 km) to the intervention with no differences between IG and CG. At the 6-month follow-up, 36/42 (86%, 95% CI 74–95%) participants completed final assessment and 19/22 (86%, 95% CI 70–100%) the intervention. The most important reasons for non-responding were work-related (12/60; 20%) and for or drop-out pandemic-related anxiety (3/6). No patient and staff member became infected with SARS-CoV-2 during the study.

**Conclusion:**

The feasibility of the MBM study was confirmed in terms of predefined recruitment and retention criteria, both despite difficult conditions (including the rural setting) and patients' fears associated with the pandemic. It was crucial to develop appropriate hygiene and safety concepts that enable chronically ill patients to participate in helpful group-based interventions even under pandemic conditions.

**Clinical trial registration:**

ClinicalTrials.gov, identifier: NCT05182645.

## Introduction

The global prevalence of inflammatory bowel disease has steadily increased within the last two decades, with an average of 1 in 200 people affected (Ng et al., 2017). Crohn's disease (CD) has an overall incidence in Germany of 6.6 new cases per 100,000 inhabitants per year, with ~25,500 patients per year who require inpatient treatment (Preiß et al., 2014; Ng et al., 2017; Sturm et al., 2022). Due to a large number of symptoms, the health-related quality of life (HRQOL) of many patients with CD is comprehensibly impaired in the most productive years of their lives. The most frequently reported symptoms with an impact on HRQOL are diarrhea, abdominal pain, fatigue, anemia, weight loss, recurrent fistulas, and extraintestinal manifestations (Romberg-Camps et al., 2010; Schirbel et al., 2010; Danese et al., 2015; Gomollón et al., 2017). Accordingly, studies have shown a statistically significant correlation between disease activity, need for retreatment, and quality of life (Casellas et al., 2000; Blondel-Kucharski et al., 2001; Bernklev et al., 2005). In addition to physical functions, emotional wellbeing, as well as social and interpersonal interactions, also play an important role in individual HRQOL. A stable social network is perceived as helpful by patients (López Blanco et al., 2005; Katz et al., 2016), while anxiety and depression, as well as dysfunctional coping with the disease, can have a negative impact on HRQOL (van der Eijk et al., 2004; Mawdsley and Rampton, 2005; Tomazoni and Benvegnú, 2018). Consequently, patients' quality of life has increasingly become focused in research on Crohn's disease and other gastrointestinal disorders (Borgaonkar and Irvine, 2000).

In line with these observations, randomized studies have shown that mind-body therapies, meditation, mindfulness, relaxation, stress management programs, and yoga may improve disease-specific quality of life and can even reduce disease-related pain in patients with IBD (Boye et al., 2011; Langhorst et al., 2013, 2015; Gerbarg et al., 2015; Neilson et al., 2016; Norton et al., 2017; Ewais et al., 2019; Torres et al., 2019). Furthermore, preliminary results from small studies suggest that patients with CD may benefit from a moderate exercise program in terms of quality of life (Ng et al., 2007), while a survey substantiated additional beneficial effects on perceived stress by exercise therapies (Torres et al., 2019). There is also some evidence that stress is associated with a higher risk of relapse in IBD (Bitton et al., 2003). In addition, Cognitive behavioral therapy (CBT) has a short-term beneficial effect on QoL in adults with IBD (Gracie et al., 2017). These first promising approaches try to explore possible psychoneuroimmunological connections between the nervous system and the immune system up to gut mucosal levels. Moreover, initial evidence also exists for further lifestyle modifications in the context of IBD (Gracie et al., 2018, 2019; Torres et al., 2019). Of particular interest is the topic of nutrition (Roda et al., 2020). Initial prospective studies have shown a substantially lower risk of later-onset CD in people following a Mediterranean diet (Khalili et al., 2020). In addition, herbal remedies are frequently used by patients as an adjunct to therapy, especially in patients with increased disease activity (Elsenbruch et al., 2005; Langhorst et al., 2005, 2007, 2015, 2020). A survey concluded that certain herbal remedies and acupuncture may reduce disease activity (Langhorst et al., 2015).

Based on these data, it is highly probable that multimodal concepts, which include mindfulness, relaxation methods, exercise, and nutrition, as well as herbal remedies, could be effective. The efficacy of such a multimodal program has already been demonstrated for patients with ulcerative colitis. In particular, there was a significant improvement in the short- and long-term quality of life and mental health (Elsenbruch et al., 2005; Langhorst et al., 2007, 2020; Labanski et al., 2020; Koch et al., 2021; Schlee et al., 2022).

However, the data available to date for patients with CD are still insufficient and some of the available studies have methodological limitations such as missing control groups, small sample size, or a too short follow-up period.

Consequently, there is a need for high-quality studies on multimodal integrative interventions. In January 2020, when recruitment was supposed to start, the first corona cases were reported in Germany.

It quickly became evident that patients with chronic diseases, in particular, that is, at higher risk for a severe COVID course, were increasingly hesitant to utilize medical treatments (Musche et al., 2020). Grunert et al. (2020) reported that patients with IBD were significantly more affected by the COVID-19 pandemic than their non-IBD peers.

Moreover, it was not until 2021 that a cross-sectional study revealed that generalized anxiety is more prevalent in rural communities, whereas COVID-19-related fear is elevated in metropoles (Diala and Muntaner, 2003; Probst et al., 2006; Schweda et al., 2021).

Taken together, we hypothesize that a holistic, comprehensive mind-body lifestyle modification program is a feasible intervention for patients with Crohn's disease even under pandemic circumstances. Therefore, the evaluation of a multimodal integrative program within the framework of this feasibility study aims on one hand to close gaps in care and on the other hand to contribute to the expansion of evidence with high methodological quality to continuously improve the treatment strategies for patients with Crohn's disease.

## Materials and methods

### Recruitment and patient characteristics

In addition to a call for studies in social and print media, we contacted a total of 700 members of the German Crohn's Disease/Ulcerative Colitis Association (DCCV e.V.)—a patient self-help association—from July 2020 to January 2021. Therefore, we considered a radius of 100 km from the study center to account for the population density in northern Bavaria (Upper Franconia) of 147 inhabitants per km^2^ (Bayerisches Landesamt für Statistik und Datenverarbeitung, 2014).

Patients who returned received detailed information about the study. The following inclusion criteria were considered: (a) patients between 18 and 75 years, (b) with a confirmed diagnosis of Crohn's disease, (c) stable medication for at least 3 months, and (d) signed informed consent. In contrast, patients with (a) a current highly acute course, (b) complete colectomy, (c) severe mental illness (e.g., major depression, addiction, and schizophrenia), (d) severe comorbid somatic diseases (e.g., diabetes mellitus, and oncological diseases), (e) pregnant women, and (f) participants of stress reduction programs or clinical studies on psychological interventions during the time of study were not eligible to participate in the study.

### Study design

To investigate the feasibility of a comprehensive mind-body lifestyle modification program in patients with Crohn's disease, we chose a prospective controlled randomized study design with four data collection points, using different data collection methods. The study was approved by the Ethics Committee of the Bavarian Medical Association (No. 19096), registered at ClinicalTrials.gov (NCT05182645), conducted according to the Declaration of Helsinki, and reported according to the CONSORT statements.

After written informed consent and baseline assessment, patients were randomized in a ratio of 1:1 to the intervention group or the control group stratified by gender, disease severity (clinical remission: mean Harvey Bradshaw Index HBI ≤ 5; mild disease HBI ≥ 6), and medication (immunomodulator yes/no) by de-aging sealed envelopes by the study management at Bamberg Hospital (out-patient department for integrative medicine) at two time points (September 2020 and January 2021). While the intervention group attended the program immediately after the first data collection point (week 0), the control group received a single psychoeducation workshop with information for self-directed application and started the full intervention 9 months later (week 36).

**Figure 1:**
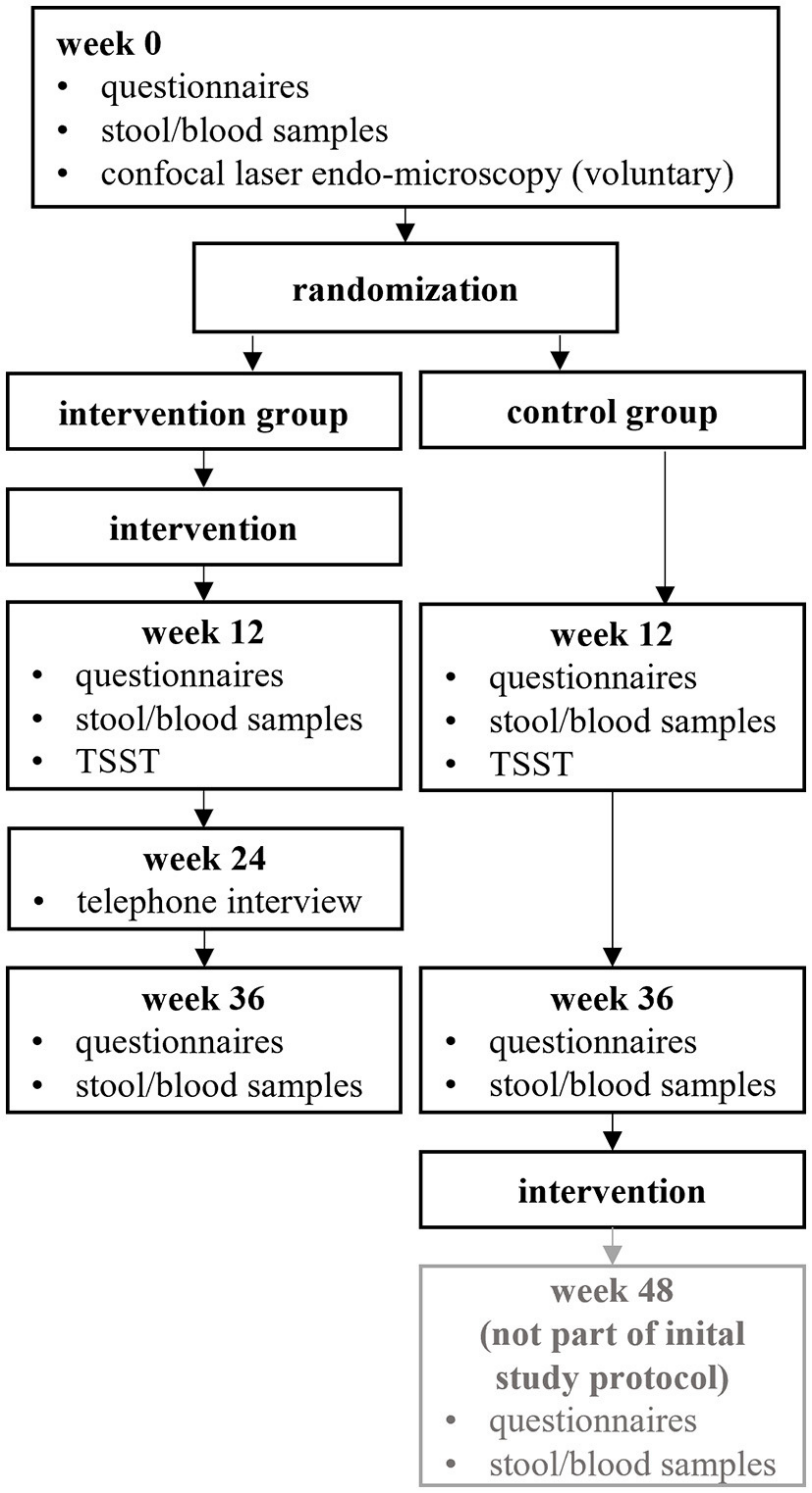
Illustration of the study design. TSST, Trier Social Stress Test.

Following the intervention (week 12) and after 6 months (week 36), the questionnaires were again administered and laboratory parameters were collected by independent members of the research department trained in good clinical practice. In addition, the Trier Social Stress Test (TSST), which is a highly reliable method of inducing acute stress, was administered at week 12. Three months after the intervention, subjects in the intervention group were asked to participate in a partially standardized guideline-based telephone interview. Subsequently, subjects in the control group had the opportunity to participate in the intervention (Figure 1). The intervention took place on the premises of the out-patient department and the study visits on the premises of the research team, both from the Department of Internal and Integrative Medicine, Sozialstiftung Bamberg, Bamberg, Germany.

### Intervention

As part of the group intervention, five to seven patients per group participated in a 60-h mind-body medicine and comprehensive lifestyle modification training program over a 10-week-period (i.e., 6 h 1 day a week for 10 weeks) from 11 a.m. to 5 p.m. An experienced mind-body instructor and an experienced gastroenterologist specializing in integrative medicine guided the sessions.

Program topics focused on different procedures described above, for which scientific evidence already exists in patients with CD in an individual setting.

This includes, in particular, stress reduction and stress management, based on the Mind-Body Medical Institutes of the Harvard Medical University (Benson and Stuart, 1993) and the Mindfulness-Based Stress-Reduction (MBSR) program of the University of Massachusetts (Kabat-Zinn, 2013) as described previously in a study with CU patients (Langhorst et al., 2007; Schlee et al., 2022). Techniques taught included relaxation, mindfulness meditation, breathing, yoga, and qi gong, but also elements of cognitive behavioral therapy (CBT) and psychoeducational approaches like stress management, coping skills training, and communication. CBT techniques included perceiving and recognizing automatic thoughts and mental distortions with a focus on one's own patterns of perception and evaluation to meet them with a non-judgmental and self-kind attitude. In addition, Mediterranean whole food nutrition, as suggested by the German consensus treatment guidelines (Sturm et al., 2022), light exercise, and walking were core elements. Complementary self-care strategies, such as hydrotherapy and herbal medicine for gastrointestinal symptoms were also demonstrated and trained. In the time between the out-patient appointments, patients were asked to fill in exercise diaries daily, were given a link to audio guides for relaxation/meditation, and a variety of print information and exercises as homework to encourage 60 min home practice daily.

Participants in the control group received care as usual and a one-time 90-min workshop on mind-body medicine and complementary self-care strategies (on site respectively online). Following the measurement at Week 36, this group was also given the opportunity to participate in the intervention.

At all appointments in attendance, strict adherence to a comprehensive hygiene concept (including adherence to distance rules the obligation to wear an FFP-2 mask throughout the day) was ensured.

### Outcomes and research aims

The endpoints focused on feasibility, investigations, and intervention can be find in Table 1. The following definitions of successful feasibility may serve as the basis for conducting a future large-scale confirmatory study:

1. Recruiting more than 10% of eligible patients who were contacted and invited to the study center,
2. at least 67% of the recruited study participants completed the intervention phase and the final follow-up assessment.

**Table 1:** P.I.C.O. model endpoints focused on study feasibility, investigations, and intervention.

Population	- Patients with Crohn's disease
Intervention	−10 week mind-body medicine and comprehensive lifestyle intervention
Control	- Control group: care as usual and one-time education of 90 min on naturopathic self-help strategies
Outcome	Feasibility of the study and investigations 1. *Recruiting success*: Proportion of individuals contacted, who °1.1) reported for the study and °1.2) were enrolled. 2. *Compliance*: Proportion of patient randomized who completed °2.1) the post-survey, °2.2) the Trier Social Stress Test, °2.3) the follow-up survey among included and randomized patients, and ° 2.4) the telephone interview among patients of the intervention group.
	Feasibility of the intervention 3. Feasibility of *intervention* included °3.1) the proportion of patients who started the study and participated in the intervention, °3.2) recording of reasons for dropping out of the study, and °3.3) for non-participation in individual sessions of the intervention, °3.4) the average number of sessions attended, °3.5) satisfaction with the intervention.
	Safety 4. Number and type of severe and mild adverse events

The outcomes 3.4 and 3.5 were not part of the initial study protocol.

### Instrument design and questionnaire

The anonymous, self-administered questionnaire was compiled based on previous studies (Elsenbruch et al., 2005; Langhorst et al., 2007, 2020; Labanski et al., 2020) and tested for comprehension on three in-patients with CD. It consisted of 174 items covering demographic characteristics, disease-, quality of life- and psychological-related factors. While the focus of this paper is the evaluation of feasibility and compliance, possible treatment effects will be presented separately. Therefore, only questionnaires relevant to this paper are presented.

Disease-specific quality of life was measured using the validated German version of the *Inflammatory Bowel Disease Questionnaire*. This widely used and validated instrument consisting of 32 items with a 7-point-Likert-scale (1 = always to 7 = never) divided into the four subscales: bowel symptoms, systemic symptoms, social function, and emotional function. The total score can vary from 32 to 224, with higher scores indicating better quality of life (Janke et al., 2006).

The Harvey-Bradshaw Index is a simplification of the Crohn's Disease Activity Index (CDAI; correlation HBI and CDAI, *r* = 0.93, *p* < 0.001) and is composed of five items. Sum score was rated as remission (0–4), mild (5–7), moderate (8–16), and severe (>16) disease activity (Harvey and Bradshaw, 1980; Irvine et al., 1994).

### Statistical analyses

Response rates and study compliance, as well as treatment adherence and drop-out rates, were calculated as the proportion of patients to whom a characteristic was applied (e.g., participation in post-survey) from the total population considered (all randomized patients) and reported as a percentage with corresponding 95% confidence interval (95% CI). Reasons for individual days of absence or study drop-out were reported qualitatively and quantitatively.

Descriptive statistics were used to report baseline characteristics, including sex, marital status, schooling, occupation, age, distance to the out-patient department, disease duration, disease activity (HBI), and quality of life (IBDQ).

The evaluation of the feasibility was not designed as a confirmatory study. We followed an exploratory approach without formal testing of hypotheses and therefore did not define a formal level of statistical significance.

## Results

### Sample description

The participants were on average 48 ± 13 years old and 67% were female. Most of them reported living in a stable relationship, having a medium level of education, and working part-time. Almost a quarter of the patients were retired due to age or an illness. At the start of the study, they had been suffering from a diagnosis of Crohn's disease for an average (standard deviation) of 20 ± 12 years, reported mild current disease symptoms (HBI: 6.0 ± 3.7), and a reduced quality of life (IBDQ: 147.1 ± 28.6). The distribution of sociodemographic factors was not considerably different between the two treatment groups (Table 2).

**Table 2:** Patient characteristics at baseline of the study and intervention related factors.

	**Total**	**Intervention**	**Control**	***p*-value**
	**(*n* = 42)**	**(*n* = 22)**	**(*n* = 20)**	
**Sociodemographic characteristics**
Gender				*p* = 0.827
Male	14 (33.3%)	7 (31.8%)	7 (35%)	
Female	28 (66.7%)	15 (68.2%)	13 (65%)	
Age (years)	47.6 (12.5)	49.0 (13.6)	46.1 (11.2)	*p* = 0.345
Relationship status				*p* = 0.260
Single	6 (14.3%)	4 (18.2%)	2 (10%)	
Married/cohabitant	34 (81%)	18 (81.8%)	16 (80%)	
Divorced/separated/widowed	2 (4.8%)	0 (0%)	2 (10%)	
Education				*p* = 0.450
No qualification	1 (2.4%)	0 (0%)	1 (5%)	
Elementary school	7 (16.7%)	5 (22.7%)	2 (10%)	
Middle school	17 (40.5%)	9 (40.9%)	8 (40%)	
(Technical-) Highschool with/without (technical-) university degree	17 (40.5 %)	8 (36.4%)	9 (45%)	
Employment				*p* = 0.394
Full-time	13 (31%)	5 (22.7%)	8 (40%)	
Part-time/occasional work	16 (38.1%)	9 (40.9%)	7 (35%)	
Retired/unemployed	9 (21.4%)	7 (31.8%)	2 (10%)	
Housewife/househusband	4 (9.5%)	1 (4.5%)	3 (15%)	
Distance hospital - home (km)	68.5 (45.1)	78.0 (54.2)	58.1 (30.5)	*p* = 0.146
**Disease related factors**
Years since initial diagnosis	20.1 (11.6)	20.0 (12.3)	20.3 (11.1)	*p* = 0.924
Disease activity (HBI)	6.0 (3.7)	6.5 (4.3)	5.4 (2.9)	*p* = 0.363
Quality of life (IBDQ)	147.1 (28.6)	141.4 (30.5)	153.4 (25.7)	*p* = 0.178
Erythrocyte sedimentation rate	14.9 (2.3)	17.4 (12.8)	12.1 (13.6)	*p* = 0.243
C-reactive protein	0.6 (0.2)	0.9 (1.1)	0.4 (0.9)	*p* = 0.118
Lactoferrin	33.2 (6.9)	44.0 (45.9)	21.9 (31.6)	*p* = 0.109
**Intervention related factors**
Satisfaction (of 10 points)	9.1 (0.9)	9.0 (0.8)	9.3 (0.9)	*p = 0.249*
Participation (of 10 sessions)	9.1 (1.4)	9.2 (1.0)	8.9 (1.8)	*p = 0.486*

### Feasibility of the study and investigations

**Figure 2:**
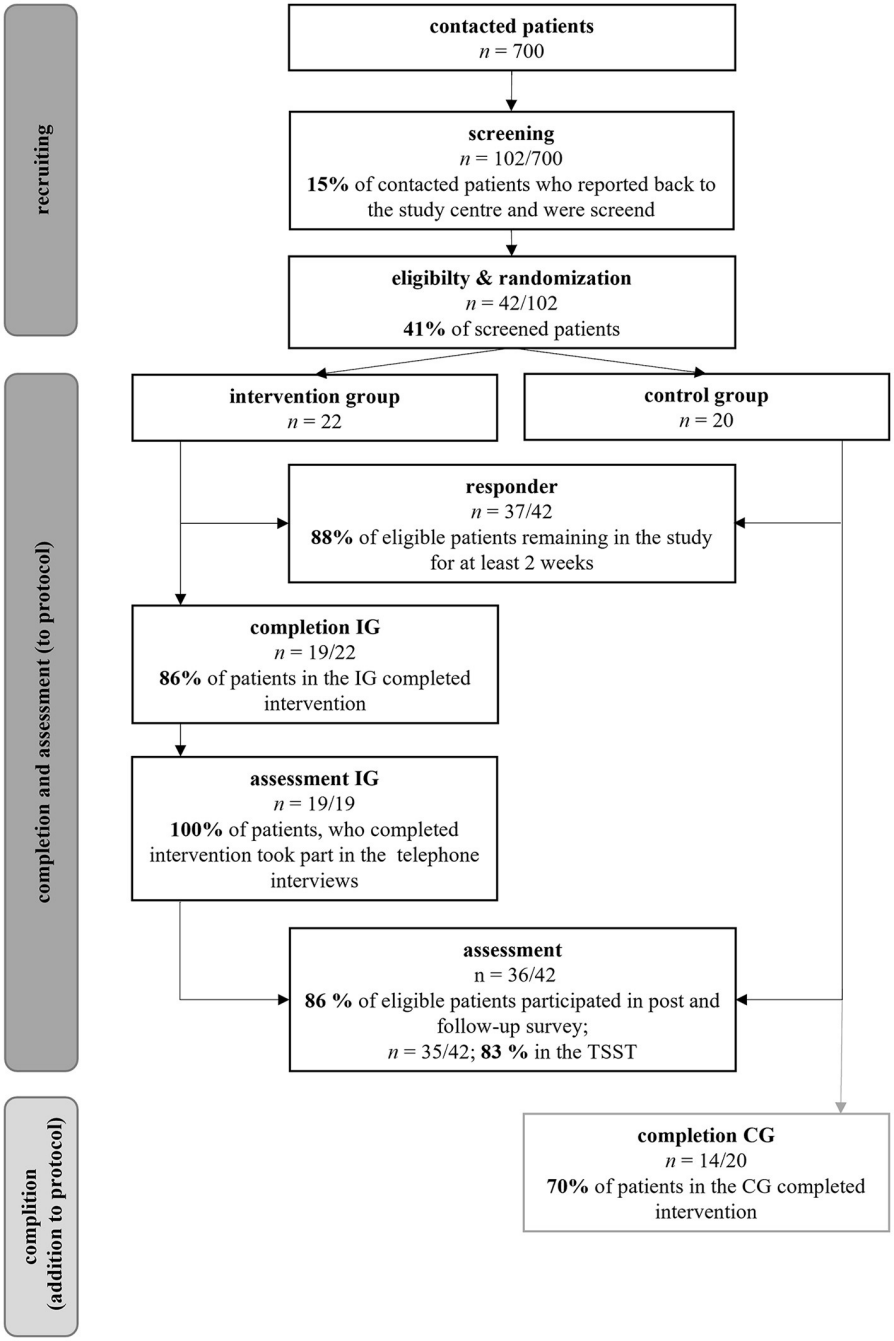
Recruitment success, compliance and feasibility of the intervention. Reasons for non-response and drop-out see Figure 3.

About 102 (=15%; 95% CI 12–17%) of 700 patients, more than the predefined 10% of the contacted patients, reported back to the study center (Figure 2). Following the screening, we randomized 6% (42/700; 95% CI 4.4–8.0) of contacted patients and 41% (42/102; 95% CI 32–50%) of patients who reported back to the study center: 22 in the intervention and 20 in the control group. Eighty-six percent (95% CI 70–100%) of the patients remained in the study until the end. The compliance within the study for the investigations at defined time points was similarly high: 86% (95% CI 70–100%) and 85% (95%-CI 67–100%) of the recruited patients completed the post and follow-up surveys, and 83% (71–95%) the Trier Social Stress Test (TSST) at Week 12. Two months after the intervention, all patients in the intervention group agreed to a partially standardized guideline-based telephone interview.

### Drop-out rates and feasibility of the intervention

**Figure 3:**
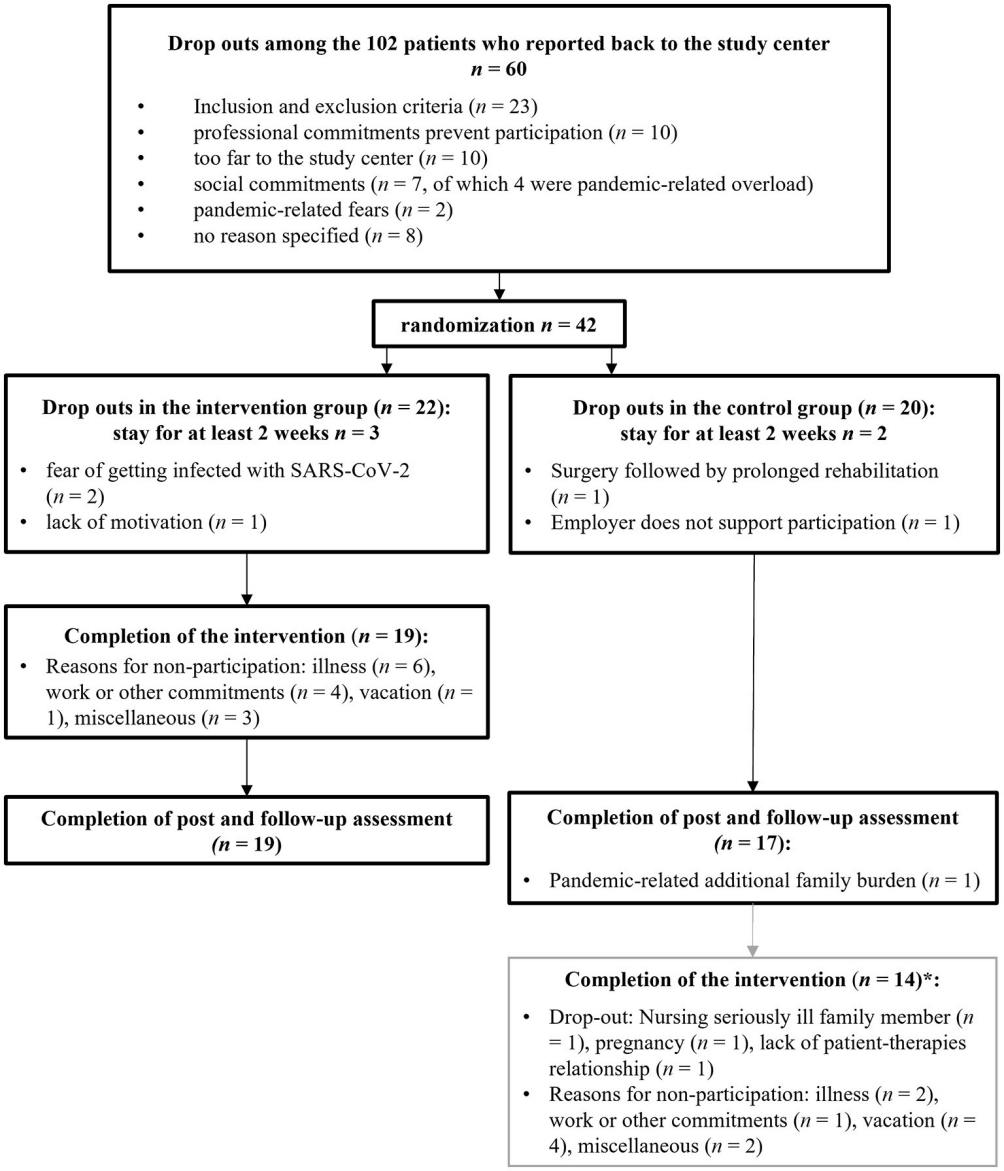
Reasons for study discontinuation. *Completion of the intervention was not part of the initial study protocol.

One-third of the patients, who contacted the study center, did not meet the inclusion criteria or met one or more exclusion criteria. Reasons for non-response are listed in Figure 3. Twelve percent of the recruited patients (95% CI 2–23%) dropped out of the study within 2 weeks (IG: 3, CG: 2). Two patients (IG) had hoped to be assigned to the spring/summer waiting control group. For them, participation in a group intervention was not an option due to the high restriction of the measures related to the pandemic (e.g., lock-down) at the time of randomization. Further reasons are reported in Figure 3. The drop-out rates did not differ (*95% CI* −0.30–0.32) between IG (14%) and CG (15%). With 86% (95% CI 70–100%), the required participation rate of 67% in the intervention group was met.

The following complementary findings are made with regard to feasibility and compliance. Of the 20 patients in the control group, 14 participated in the intervention (70%, 95% CI 48–90%). Patients participated in an average of 9.1 ± 1.4 of the 10 sessions, with no differences between the two groups (*95% CI* −0.67–1.40). Symptoms of a common cold were reported more frequently in the intervention group (winter months) and vacation more often in the control group (summer months). Patients covered an average distance of 71.5 ± 48.7 km to the intervention (25%-percentile: 37 km; 50%-percentile: 59 km, 75%-percentile: 103 km). The longest distance traveled one way was 227 km. However, patients with a further journey were more likely to skip a session (*95% CI* −0.66 to −0.04).

The satisfaction with the intervention was high at 9.1 ± 0.9 (*95% CI* −0.99–0.27), out of a possible 10 points (best score).

### Safety of the study under pandemic conditions

During the entire study period, two severe (SAE) and four mild adverse events occurred, which were presumably not causally related to the study. One SAE patient in the control group developed an episode of her Crohn's disease 2 months after baseline measurement and was admitted to the hospital as an inpatient. The second SAE patient suffered pancreatitis after elective endoscopic retrograde cholangiopancreatography (ERCP) shortly after baseline measurement, which kept her from participating in the intervention for the first 2 days of intervention. The four mild adverse events all occurred in the intervention group. For safety reasons, these patients with symptoms typical of a respiratory infection remained at home to avoid infecting other group members in case of (corona) virus infection. Due to this respectful interaction of the group members in the pandemic, as well as the high hygiene standards, no patient and no member of the therapeutic or scientific team became infected with SARS-CoV-2.

## Discussion

This paper provides three findings we believe to be important. This study proves for the first time the feasibility of a randomized controlled trial with a long observation period and a comprehensive mind-body lifestyle modification program in patients with Crohn's disease. As the recruitment rate and compliance were achieved according to predefined criteria, the feasibility of such a trial is also given in rural regions and under pandemic conditions. Second, a high level of adherence to the program appointments could be demonstrated. Third, a high level of patient satisfaction with the multi-modal intervention was shown, although this required a strong and continuous commitment.

### Feasibility of the study

A particular challenge with regard to the feasibility of studies for patients with chronic diseases, such as CD, is the pandemic. A large cross-sectional German study including almost 17,000 participants could demonstrate elevated levels of generalized anxiety, COVID-19-related fear, adherent/dysfunctional safety behavior, and subjective risk perception in participants with high-risk conditions, such as diabetes or conditions of immunodeficiency (Kohler et al., 2021). In consequence, rates of mental distress and disorders increased significantly (Bäuerle et al., 2020; Mehrotra et al., 2020). Until a vaccine was developed, it was therefore recommended that risk groups, in particular, isolate themselves. However, studies have clearly shown that the COVID-19 pandemic has led to a decrease in the utilization of many medical care services (Mehrotra et al., 2020). This can be reported particularly for chronic conditions (Hacker et al., 2021). For example, in the first summer of the pandemic, 4 in 10 adults surveyed reported that they had postponed or avoided routine or emergency care because of the pandemic (Czeisler et al., 2020). Therefore, treatment teams increasingly focused on transferring face-to-face group therapy to online group chats during the early months of the COVID-19 pandemic (Scholl et al., 2021). However, since multimodal concepts can only be partially adapted to a virtual format, the next challenge was now to develop appropriate hygiene and safety concepts that enable chronically ill patients to participate in helpful programs even under pandemic conditions and to feel safe and in good hands. Even before the S-3 guideline supplement on the COVID-19 pandemic for patients with inflammatory bowel disease was published in October 2020 (Stallmach et al., 2020), the Bamberg study team, therefore, decided to start recruitment.

Against this background, the unexpectedly high interest of the contacted patients with 15% contacting the study center is remarkable. On one hand, this could be due to the many years of very good cooperation with the national patient organization for IBD (“Deutsche Morbus Crohn/Colitis Ulcerosa Vereinigung,” DCCV e.V.), whose members were probably familiar with the expertise and commitment of the study center. Moreover, the role of the self-help group in studies could be very important, as members trust their patient organization, which communicates respectfully with its members and provides information to the best of its knowledge. On the other hand, it could also have played an important role to convey the mitigation efforts to ensure that this mind-body medicine program is safe [e.g., small groups (*n*_*max*_ = 7), large rooms, regularly ventilate, mask requirements, and social distancing] and to explain clearly how to safely access care in the invitation to participate.

If we look at the patients who contacted the study center but then decided against participation before the randomization, it is noticeable that, in particular, professional or social commitments were the most frequently cited reasons for deciding against participation, in addition to a perceived too far journey. This is in line with the results of the recently published qualitative study on patients with ulcerative colitis (Schlee et al., 2022). Less than 4% of eligible patients decided not to participate due to pandemic concerns or fears, which again may indicate that patients felt safe in the study setting presented.

The feasibility of the study was also quantified by the compliance within the study, which was determined as the proportion of subjects who completed the scheduled assessments at different time points. Eighty-six percent of randomized patients completed both the post-survey directly after the intervention and the 6-month follow-up. This figure is comparable to other studies (Berrill et al., 2014; Neilson et al., 2016). However, these studies were not conducted under pandemic conditions, which must be considered.

In addition, 83% of the participants in both study groups carried out the Trier Social Stress Test (TSST), and all patients in the intervention group agreed to a partially standardized guideline-based telephone interview 2 months after the intervention. The fact that patients were even willing to undergo a stress-triggering test, which tends to be perceived as rather unpleasant, as well as a time-intensive telephone interview, speaks for a very good identification with the study and strong patient commitments.

In the control group, only three drop-outs were recorded after randomization, most of which could not be related to study design or waiting time. The fact that, at the end of the actual study, 14 of the 17 patients remaining in the control arm also took the opportunity to participate in an intervention suggests that a multimodal comprehensive mind-body lifestyle modification program addresses the unmet needs of the patients. Moreover, the one-time 90-min workshop on mind-body medicine and complementary self-care strategies in the control group might have been a motivation to stay.

### Feasibility of the intervention

To examine the feasibility of the stress-management and comprehensive lifestyle-modification program, we studied patient adherence to appointments with a focus on reasons for non-participation in individual sessions and for dropping out of the study. In addition, we asked the participants about their satisfaction with the intervention.

Three of five patients, who terminated the study early, dropped out in the first 2 weeks after randomization. Although more patients in the intervention group dropped out of the study directly, verbally reported back dissatisfaction with the outcome of randomization was higher in the control group, which may reflect patients' need for a multimodal, multi-week approach. Even though long observation periods are important from a scientific point of view and the patients in the control group received a single psychoeducation workshop, the patients' desire to be allowed to participate in a potentially helpful intervention in a timely manner is understandable.

Because studies have shown that non-compliance is a barrier to learning mindfulness, which is a key element in the investigated comprehensive mind-body lifestyle modification program, it is especially important to examine and understand the reasons for drop-outs and lack of compliance after the start of the intervention (Lymeus et al., 2019; Zhang et al., 2021). Unfortunately, drop-out rates of 25% or higher have been reported in representative studies (Abbott et al., 2014; Lamothe et al., 2016). Adherence to mindfulness-based interventions in patients with inflammatory bowel disease in randomized trials published to date ranged from 55% (Schoultz et al., 2015, 2016) to 58% (Berrill et al., 2014) and remained below the values achieved here even in a study with free group choice (82%) (Neilson et al., 2016).

Adherence from randomization to the end of the holistic integrative medicine intervention was 86%, the same as in a meta-analysis for yoga interventions in Europe (Cramer et al., 2016). However, compared to study data showing that drop-out rates increase with underlying medical conditions and even nearly double as the number of sessions increases (under 8 vs. over 12 sessions) (Cramer et al., 2016), adherence in the context of this study appears unexpectedly high, even more so under pandemic conditions. In this study, although the number of face-to-face sessions is in the middle range at 10 weeks, individual yoga sessions, as in the meta-analysis mentioned above, were considerably shorter at 1–2 h than a duration of 6 h in the out-patient department.

Despite the comprehensive hygiene concept of the hospital, SARS-CoV-2-related fears or anticipated pandemic-related challenges were a major issue for patients already in the recruitment phase and were responsible for half of the study drop-outs in the further course (*n* = 3 out of 6). On the other hand, this study showed that Crohn's disease patients are motivated to participate in a comprehensive mind-body lifestyle modification program even under pandemic conditions and that its implementation is possible under high hygiene standards without endangering the health of the partly immunosuppressed participants. Not a single SARS-CoV-2 positive patient or therapist during the intervention periods was recorded. In addition, virtual sessions may not appear to be an equivalent alternative to face-to-face sessions. In a systematic review of Internet-based interventions focusing on mindfulness, adherence ranged from 38 to 78% (mean 40%) (Christensen et al., 2009), well below the adherence in the present study.

The patients participated very regularly in the group intervention itself and were extremely satisfied, although a high degree of cooperation (including no sick leave possible) was also required outside the program. The high participation rate is particularly remarkable because many patients travel long distances to take part. To relieve the patients and to make it easier to reconcile the intervention with work, it would be of central importance here to have the intervention recognized as a health insurance benefit with the consequence of the possibility of issuing a certificate of incapacity for work. This is particularly important because a program for building-up personal health competence could be interesting not only for the patients' quality of life but also from an economic point of view.

The results must be interpreted carefully considering several potential limitations. First, a weakness is the small case number, although this is in the nature of feasibility studies. Second, a self-selection bias is likely due to the voluntary character of the intervention and the overrepresentation of women. For example, studies concluded that women use different coping strategies than men and that emotional coping strategies, in particular, may play a greater role. Women might therefore have felt more addressed by the holistic approach (Sarid et al., 2017). Furthermore, it is possible that individuals with an interest in integrative medicine, naturopathy, and/or mindfulness-based exercises, as well as patients who are motivated for group interventions, in general, were more likely to contact the study center. The strengths of the study are the high-quality study design, the data assessment by “good-clinical-practice” certified researcher, and the discussion of drop-out rates and reasons against the background of planning and feasibility of studies under challenging conditions (rural region and pandemic). Where appropriate and necessary (e.g., assessment of disease activity and quality of life), standardized, established, and validated questionnaires were used to allow meaningful classification and, if necessary, subsequent comparison of results in reviews and meta-analyses. Both survey waves, starting September 2020 and January 2021, were conducted identically and strictly according to the study protocol.

## Conclusion

In summary, an expansion of the offer of comprehensive mind-body lifestyle modification programs is desirable for patients with Crohn's disease, since this offer is very well accepted by the patients even under rural and pandemic circumstances. Patients showed high adherence, were highly satisfied with the intervention, traveled long distances to the out-patient department, and even participated without a sick leave certificate for the days of the study.

## Data availability statement

The raw data supporting the conclusions of this article will be made available by the authors, without undue reservation.

## Ethics statement

The studies involving human participants were reviewed and approved by Ethics Committee of the Bavarian Medical Association: No. 19096. The patients/participants provided their written informed consent to participate in this study.

## Author contributions

JL and TK contributed to the conception and design of the study. NB, CU, ÖÖ, and JL managed the project administration and the data collection. NB performed the statistical analysis. NB and CL wrote the first draft of the manuscript. All authors have reviewed and edited the manuscript and agreed to the submitted version.

## Funding

This study was supported by the Bavarian State Ministry for Health and Care (Germany) by means of the funding program Gesund.Leben.Bayern (in English: Healthy.Living.Bavaria), Reference Number GE7-2497-GLB-19-V4. The sponsors of the present study had no role in the design, execution, interpretation, or writing of the study.

## Conflict of interest

CL received lecture fees from Celgene GmbH, Roche GmbH, Novartis Pharma GmbH, BMS GmbH & Co. KGaA, Mundipharma GmbH Co. KG, Merck KGaA. JL was a speaker for Repha GmbH, Techlab Inc., Falk Foundation, Takeda, Celegene GmbH and Willmar Schwabe and received research funding from Repha GmbH, Techlab Inc., Falk Foundation and Willmar Schwabe. The remaining authors declare that the research was conducted in the absence of any commercial or financial relationships that could be construed as a potential conflict of interest. The handling editor SS declared a shared affiliation with the author TK at the time of review.

## Publisher's note

All claims expressed in this article are solely those of the authors and do not necessarily represent those of their affiliated organizations, or those of the publisher, the editors and the reviewers. Any product that may be evaluated in this article, or claim that may be made by its manufacturer, is not guaranteed or endorsed by the publisher.
